# Leptin, adiponectin and serotonin levels in lean and obese dogs

**DOI:** 10.1186/1746-6148-10-113

**Published:** 2014-05-13

**Authors:** Hyung-Jin Park, Sang-Eun Lee, Jung-Hyun Oh, Kyoung-Won Seo, Kun-Ho Song

**Affiliations:** 1 Laboratory of Veterinary Internal Medicine, College of Veterinary Medicine, Chungnam National University, Daejeon 305-764, South Korea; 2 Division of Malaria & Parasitic Diseases, Korea National Institute of Health, Korea Centers for Disease Control and Prevention, Osong-eup, Cheongwon-gun, Chungbuk 363-951, South Korea; 3 Department of Statistics, Chung-Ang University, Seoul 156-756, South Korea

**Keywords:** Adiponectin, Leptin, Obese dog, Serotonin

## Abstract

**Background:**

Serotonin (5-hydroytryptamine or 5HT) is associated with numerous behavioral and psychological factors and is a biochemical marker of mood. 5HT is involved in the hypothalamic regulation of energy consumption. 5HT controls appetite in the central nerve system (CNS) and stimulates intestinal mobility. There are few studies looking at the role of 5HT and the relationship between peripheral circulating serotonin and obesity. The aim of this study was to find any differences in leptin, adiponectin, and 5HT between lean and obese dogs and to identify correlations among these factors.

**Results:**

Leptin, triglyceride (TG) and cholesterol levels were higher in the obese group (all p *<* 0.01). Adiponectin and 5HT levels were higher in the lean group compared to the obese group (p < 0.01). Leptin (*r* = 0.628, p *<* 0.01), TG (*r* = 0.491, p *<* 0.01) and cholesterol (*r* = 0.419, p < 0.01) were positively correlated with body condition score (BCS), and adiponectin (*r* = -0.446, p < 0.01) and 5HT (*r* = -0.490, p *<* 0.01) were negatively correlated with BCS. Leptin was negatively correlated with adiponectin (*r* = -0.294, p *<* 0.01) and 5HT (*r* = -0.343, p *<* 0.01). 5HT was negatively correlated with leptin (*r* = -0.343, p *<* 0.01), TG (*r* = -0.268, p *<* 0.05) and cholesterol (*r* = -0.357, p *<* 0.05).

**Conclusions:**

5HT is an important appetite control neurotransmitter, but there are limited studies for 5HT levels related to obesity in dogs. To the best of our knowledge, this is the first study to evaluate peripheral 5HT levels in obese dogs. From this research, we can assume that 5HT may be correlated with canine obesity. Further studies will be needed to further elucidate the role of low serum 5HT levels in canine obesity.

## Background

Obesity is a disease characterized by excessive adipose tissue accumulation in the body. It is a common nutritional problem in small animal medicine [1,2]. Ideally, fat mass accounts for approximately 15% to 20% in dogs and cats [3,4]. Pets are considered overweight when body weight exceeds ideal body weight by 10% to 20%, and pets are considered obese when body weight exceeds ideal body weight by 20% to 30% [4]. Worldwide, approximately 25% to 35% of adult cats and 35% to 40% of adult dogs are overweight or obese [5,6]. Middle-aged neutered male cats and middle-aged spayed female dogs are at the highest risk for obesity [7]. According to Lund et al. [8], some purebred dogs including Shetland Sheepdogs, Golden Retrievers, Dachsunds, Cocker Spaniels, and Rottweilers have a higher risk of obesity than other breeds. Canine obesity is associated with several health conditions such as insulin resistance, pancreatitis, cruciate ligament rupture, lower urinary tract disease, oral disease, neoplasia, osteoarthritis and decreased longevity [9,10]. Additionally, obesity could be risk factors for brachycephalic syndrome and musculoskeletal problems and it may increase risks associated with anesthesia [7]. Leptin was the first identified adipokine and has a role of regulating body fat mass through appetite control and increased energy metabolism [11]. Restriction of food intake results in a suppression of leptin levels, which is returned by refeeding [11]. Circulating leptin levels correlates positively with adipose tissue mass and exogenous leptin replacement decreases fast-induced hyperphagia [11]. Circulating leptin interacts with peripheral 5HT and decreases appetite [3]. The majority of obese animals and humans have high serum leptin levels, which suggests leptin resistance [11]. Leptin resistance results from a signaling defect in leptin-responsive hypothalamic neurons and impaired transport into the brain [11]. Obesity can be a cause of leptin resistance, but a lack of sensitivity to circulating leptin may also induce obesity [11,12]. Adiponectin is believed to be produced by mature adipocytes and it contributes to increased insulin sensitivity, decreased blood glucose, and decreased tissue triglyceride (TG) content in the liver and muscle [12]. Related to obesity, adiponectin has a negative correlation with obesity [12]. The decreasing of adiponectin in obesity is more severe with visceral than subcutaneous adiposity in human and the composition of adiponectin is also changed. Increased production of pro-inflammatory cytokines such as TNF-α and IL-6 with increased fat mass may inhibit adiponectin gene expression [12]. Serotonin (5-hydroytryptamine or 5HT) is associated with numerous behavioral and psychological factors and is a biochemical marker of mood [13,14]. 5HT is involved in the hypothalamic regulation of energy consumption and 5HT levels in the central nervous system (CNS) are influenced by energy conditions [15]. In the CNS, 5HT has a hypophagic effect owing to an inhibitory interaction with the orexigenic system through hypocretins and neuropeptide Y (NPY). 5HT also likely has a stimulatory effect on the anorexigenic melanocortin system [16]. Concerning interactions among leptin and 5HT, a previous mouse model study reported that peripheral leptin levels were reduced by 5HT, and 5HT exerted a direct effect on adipocytes and regulated leptin release from adipocytes [15]. Few studies have been published looking at the role of 5HT and the relationship between peripheral circulating serotonin and obesity [14,15]. There have been no studies performed to evaluate peripheral 5HT levels in obese dogs until now. The aim of this study was to identify differences and correlations among peripheral concentrations of leptin, adiponectin, and 5HT between lean and naturally obese dogs.

## Methods

### Ethics statement

Blood samples of dogs owned by private individuals were used in the present study. The sample collection protocol was approved by Laboratory Animals of Chungnam National University committee (approved No. CNU 00370) and the investigators adhered to the Guide for the Care and Use of Laboratory Animals of Chungnam National University.

### Animals and sampling

A total of 100 healthy dogs (age range: 1 to 15 years-old; mean age: 7 years) owned by clients of Chungnam National University Veterinary Medicine Teaching Hospital were recruited to be in the study from March 2012 to February 2013. Before examination, informed client consent was obtained and we adhered to a high standard of veterinary care. Owners were asked to have their dogs fast for 12 hours prior to presentation for blood sampling. One investigator weighed and examined each dog to screen for potential underlying inflammatory conditions. The same investigator determined a body condition score (BCS) and assigned the dog to either the control group (BCS 4-5/9) or the obese group (BCS 7.5-9/9) utilizing a 9-point BCS system [17]. A screening test for overall health included a physical examination, along with history taking, complete blood count (CBC), serum biochemistry, electrolyte analysis, total thyroxine (tT4), and adrenocorticotropic hormone (ACTH) assay. After the screening test, 18 dogs were excluded in this study due to underlying disease including liver failure, heart disease and hyperadrenocorticism. Also, dogs that were receiving serotonergic drugs, such as fluoxetine or being fed serotonin-containing food, such as bananas, tomatoes, pineapples, or walnuts were excluded from this study. Finally, 82 healthy dogs were enrolled for this research (Table 1).

**Table 1:** Profile of dogs in lean and obese groups (Mean ± SE)

	**Age**	**Body weight**	**BCS**	**Gender**	**Breed**
Lean group	4.12 ± 2.77	5.47 ± 0.23	4.32 ± 0.07	Male (4)	Shih-Tzu (5)
				Male	Poodle (3)
				Castrated (9)	Maltese (3)
				Female (13)	Mongrel (12)
				Female spayed (15)	Schnauzer (3)
					Cocker spaniel (1)
					Yorkshire terrier (1)
					Beagle (13)
Obese group	7.07 ± 3.20	10.73 ± 1.25	8.09 ± 0.09	Male (1)	French bulldog (1)
				Male	Shih-Tzu (13)
				Castrated (18)	Cocker spaniel (5)
				Female (10)	Poodle (3)
				Female spayed (12)	Mongrel (2)
					Golden retriever (2)
					Yorkshire terrier (4)
					Beagle (2)
					Maltese (4)
					Dachshund (1)
					Jindo (1)
					Chihuahua (1)

### Sample analysis

For adipokine analyses, we collected blood by venipuncture using minimal restraint from the jugular vein of dogs that were deemed healthy based on history and physical examination. A fraction of the collected blood was placed in an EDTA tube for a CBC. The remaining blood was allowed to clot at room temperature for 30 minutes and then centrifuged at 2,500 rpm for 15 minutes. Serum and plasma samples were frozen and stored at -80°C, and until analysis of adipokines, 5HT, tT4, cortisol, TG and cholesterol levels.

### Sandwich enzyme-linked immunosorbent assay and hormone assay

Serum leptin and adiponectin levels were measured according to the manufacturer’s protocol using a canine leptin commercial sandwich enzyme-linked immunosorbent assay (ELISA) kit (Millipore, Billerica, MA, USA) and canine adiponectin commercial ELISA kit (Milipore, Billerica, MA, USA). All samples were tested in duplicate. Serum tT4 and cortisol levels were quantified using an Immulite 1000 immunoassay analyzer (Siemens Medical Diagnostics, Los Angeles, CA, USA). Plasma 5HT concentrations were measured by a commercially available ELISA kit (Serotonin EIA Kit, Enzo Life Sciences Inc, MI, USA) according to the manufacturer’s instructions [18].

### Statistical analysis

Statistical analyses were performed by a commercially available computer-based software program (IBM SPSS Statistics 20.0.0, SPSS Inc., Chicago, IL USA). Age, body weight, and BCS results are presented as means with a standard error. Serum leptin, adiponectin, 5HT, TG, cholesterol, tT4, and cortisol levels are presented as means with a standard error and 95% confidence intercal. A *P* value of < 0.05 was considered significant. Age, BCS, leptin, adiponectin, 5HT, total thyroxine (tT4), cortisol, platelets, TG and cholesterol were compared between the lean group and the obese group using the independent *t*-test. The correlation between variables including age, sex, platelets, leptin, adiponectin, 5HT, TG, cholesterol, tT4, cortisol, and BCS were assessed by Pearson’s correlation coefficients.

## Results

### Leptin, adiponectin and 5HT levels in the lean and obese groups

**Figure 1:**
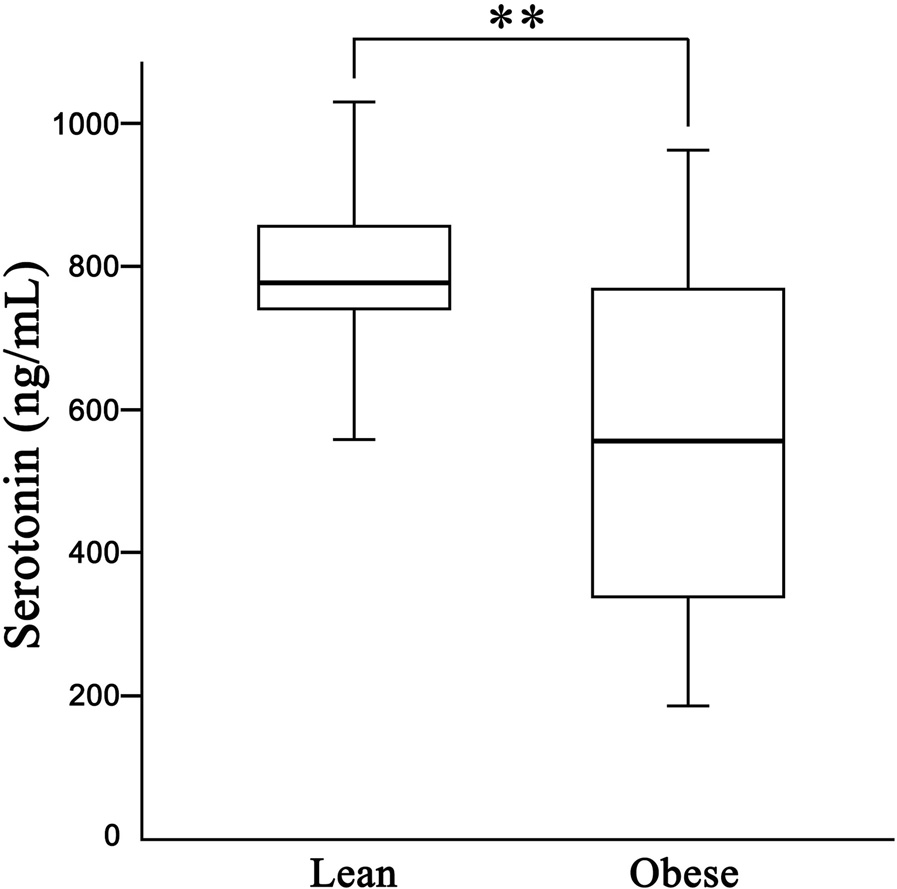
**Serotonin concentration between lean and obese groups.** Data are presented as boxes and whiskers. Each box includes the interquartile range, whereas the line within a box represents the median, and the whiskers represent the range, extending to a maximum of 1.5 times the interquartile range. Serotonin levels were significantly lower in obese group compared with that of lean group. **p < 0.01: significant difference were observed between lean and obese groups.

In this study, dogs were separated by BCS into lean and obese groups. Each group had 41 dogs. In the lean group (male, n = 13; female, n = 28), the average age was 4.12 years, the average body weight was 5.47 kg, and the average BCS was 4.32. In the obese group (male, n = 19; female, n = 22), the average age was 7.07 years, the average body weight was 10.73 kg, and the average BCS was 8.09. The distributions of BCS and gender in each group are presented in Table 1. Obesity-related parameters, including leptin, adiponectin and 5HT levels, are presented in Table 2. The obese group showed significantly higher body weights and BCS (both p < 0.01). Leptin, TG, and cholesterol levels were higher in the obese group than in the lean group (p < 0.01). Adiponectin and 5HT levels were higher in the lean group than the obese group (p < 0.01). The distribution of plasma 5HT concentration is shown in Figure 1. No significant differences were found in tT4 or cortisol between the lean and obese groups. To evaluate the effect of sex or being castrated/spayed, we divided animals intact and castrated or spayed within the lean and obese groups. In the obese group, spayed females had a lower level of 5HT than the intact female group (Table 3). The general tendency of the obesity related parameter was not influenced by gender status (Table 3). To estimate the effect of aging on obesity related parameters, the dogs were divided into two groups (young: < 8 years old; old: > 8 years old) and within each group, we created two subgroups (lean vs. obese group). Regardless of age, the lean group showed a lower level of BCS and leptin, and a higher level of 5HT than the obese group (Table 4).

**Table 2:** Obesity parameter, adipokines (leptin and adiponectin) and serotonin levels between lean and obese groups (Mean ± SE, 95% confidence interval)

	**Lean group**		**Obese group**		**P** ***-*** **value**
	**(N = 41)**		**(N = 41)**		
	**Mean ± SE**	**95% CI**	**Mean ± SE**	**95% CI**	
BCS	4.31 ± 0.07	4.16 4.45	8.07 ± 0.09	7.89 8.25	< 0.01
Leptin (ng/mL)	2.69 ± 1.08	1.91 3.46	10.27 ± 1.20	7.91 12.63	< 0.01
Adiponeptin (μg/mL)	12.18 ± 1.09	10.05 14.31	6.23 ± 0.79	4.66 7.78	< 0.01
Serotonin (ng/mL)	792.96 ± 102.45	749.49 839.47	560.43 ± 245.95	491.28 623.19	< 0.01
Triglyceride (mg/dL)	51.53 ± 29.03	41.78 61.28	138.64 ± 125.46	92.13 180.95	< 0.01
Cholesterol (mg/dL)	211.03 ± 73.80	186.23 235.82	289.97 ± 112.93	254.86 325.88	0.01
Total T4 (μg/dL)	1.74 ± 0.66	1.50 1.97	1.55 ± 0.55	1.36 1.726	0.19
Cortisol (μg/dL)	4.61 ± 2.36	3.78 5.44	5.39 ± 3.43	4.26 6.52	0.29

CI; confidence interval.

**Table 3:** Obesity parameter, adipokines, and serotonin levels according to sex (Mean ± SE)

		**Males**		**Females**	
		**Intact**	**Castrated**	**Intact**	**Spayed**
		**(N = 4)**	**(N = 9)**	**(N = 13)**	**(N = 15)**
**Lean group**	BCS	4.25 ± 0.25	4.44 ± 0.17	4.0 ± 0	4.53 ± 0.13
	Leptin (ng/mL)	5.06 ± 2.85	2.60 ± 0.48	1.59 ± 0.18	2.88 ± 0.68
	Adiponectin (μg/mL)	10.53 ± 1.29	11.55 ± 2.20	14.31 ± 2.61	11.14 ± 1.42
	Serotonin (ng/mL)	871.31 ± 61.31	736.0 ± 43.98	739.49 ± 15.69	832.21 ± 22.45
	Triglyceride (mg/dL)	64.75 ± 18.32	42.0 ± 9.54	38.57 ± 7.08	51.21 ± 5.44
	Cholesterol (mg/dL)	282.0 ± 38.02	218.33 ± 27.36	163.14 ± 18.02	204.57 ± 17.45
		**Intact**	**Castrated**	**Intact**	**Spayed**
		**(N = 1)**	**(N = 18)**	**(N = 10)**	**(N = 12)**
**Obese group**	BCS	8	8.16 ± 0.14^★★^	8.10 ± 0.23^★★^	7.67 ± 0.22^★★^
	Leptin (ng/mL)	11.6	11.66 ± 1.70^★★^	9.91 ± 3.20^★★^	8.39 ± 1.91^★★^
	Adiponectin (μg/mL)	5.2	5.92 ± 1.07^★^	6.80 ± 1.38^★★^	6.29 ± 1.99^★^
	Serotonin (ng/mL)	622.45	533.26 ± 58.89^★★^	682.14 ± 59.95	478.0 ± 74.06^*,★★^
	Triglyceride (mg/dL)	288	154.16 ± 35.13^★^	148.57 ± 45.48^★^	95.91 ± 14.08^★^
	Cholesterol (mg/dL)	325	301.38 ± 23.22^★^	240.20 ± 24.14^★^	296.72 ± 49.91^★^

^*^p *<* 0.05 between intact and spayed groups.

^★^p *<*0.05 between lean and obese groups.

^★★^p *<*0.01 between lean and obese groups.

**Table 4:** Obesity parameter, adipokines and serotonin levels according to age (Mean ± SE)

	**Young group (< 8-year old) (N = 58)**			**Old group (> 8-year old) (N = 24)**		
	**Lean group**	**Obese group**	**P- value**	**Lean group**	**Obese group**	**P- value**
	**(N = 36)**	**(N = 22)**		**(N = 5)**	**(N = 19)**	
BCS	4.31 ± 0.08	8.09 ± 0.15	< 0.01	4.40 ± 0.24	7.89 ± 0.16	< 0.01
Leptin (ng/mL)	2.64 ± 0.44	10.29 ± 1.87	< 0.01	3.02 ± 0.66	10.24 ± 1.47	0.023
Adiponeptin (μg/mL)	12.28 ± 1.19	5.83 ± 0.90	< 0.01	11.47 ± 2.66	6.68 ± 1.39	0.13
Serotonin (ng/mL)	794.70 ± 16.76	587.71 ± 54.94	< 0.01	780.41 ± 57.01	528.83 ± 53.80	0.032
Triglyceride (mg/dL)	48.21 ± 4.41	150.56 ± 34.27	< 0.01	70.80 ± 22.19	131.66 ± 26.08	0.251
Cholesterol (mg/dL)	205.97 ± 14.34	276.65 ± 19.72	< 0.01	240.40 ± 19.50	299.68 ± 31.85	0.363
Total T4 (μg/dL)	1.82 ± 0.12	1.50 ± 0.13	0.103	1.38 ± 0.17	1.58 ± 0.13	0.434
Cortisol (μg/dL)	4.42 ± 0.42	4.80 ± 0.83	0.652	5.54 ± 1.17	5.97 ± 0.78	0.793

### Correlation between variables

**Figure 2:**
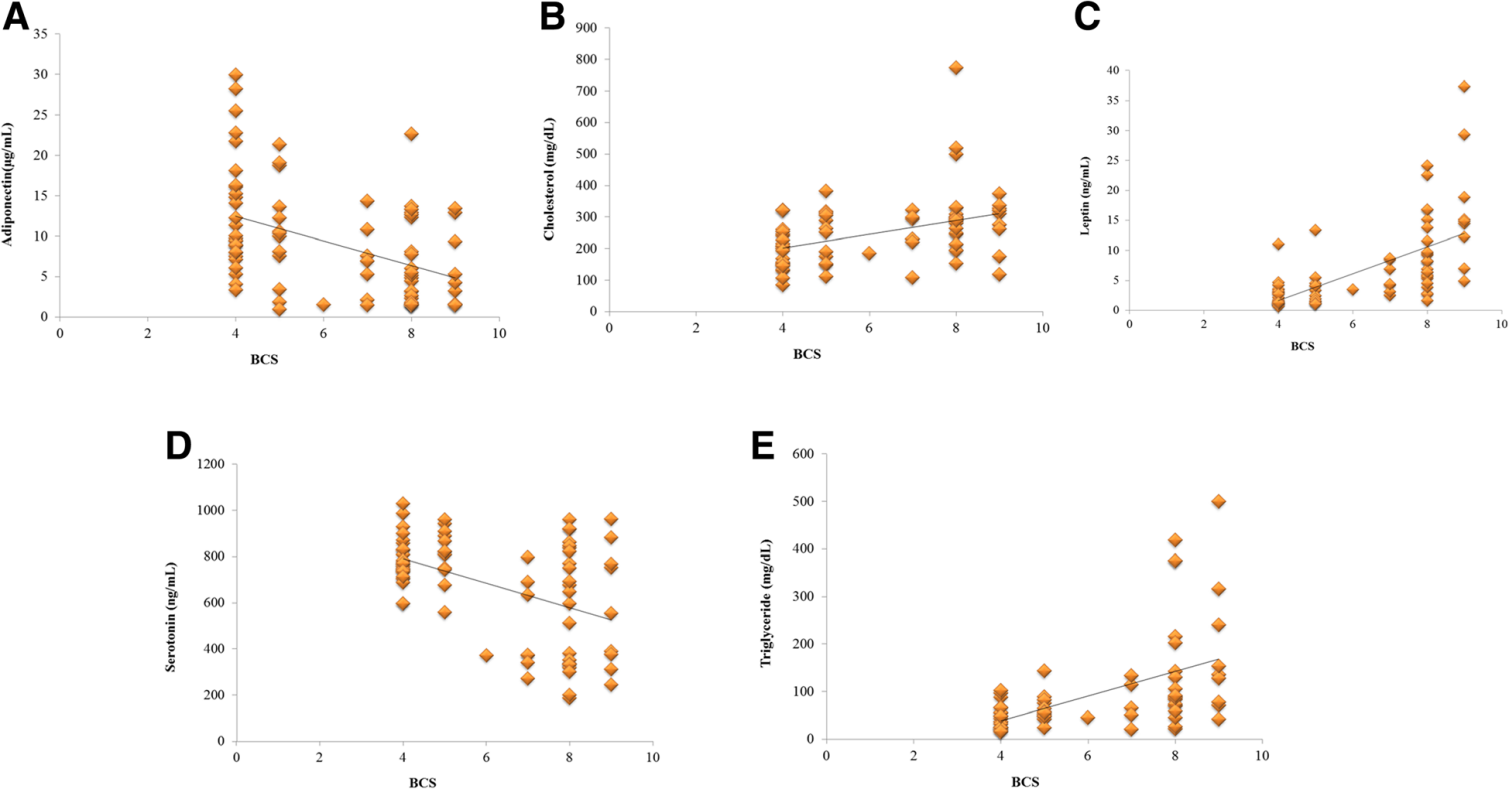
**Correlation between BCS and obesity parameters. (A)** BCS and adiponectin showed negative correlation (*r* = -0.446, p < 0.01). **(B)** BCS and Cholesterol showed positive correlation (*r* = 0.419, p < 0.01). **(C)** BCS and leptin showed positive correlation (*r* = 0.628, p < 0.01). **(D)** BCS and serotonin showed negative correlation (*r* = -0.490, p < 0.01). **(E)** BCS and tirglyceride showed positive correlation (*r* = 0.491, p < 0.01).

**Figure 3:**
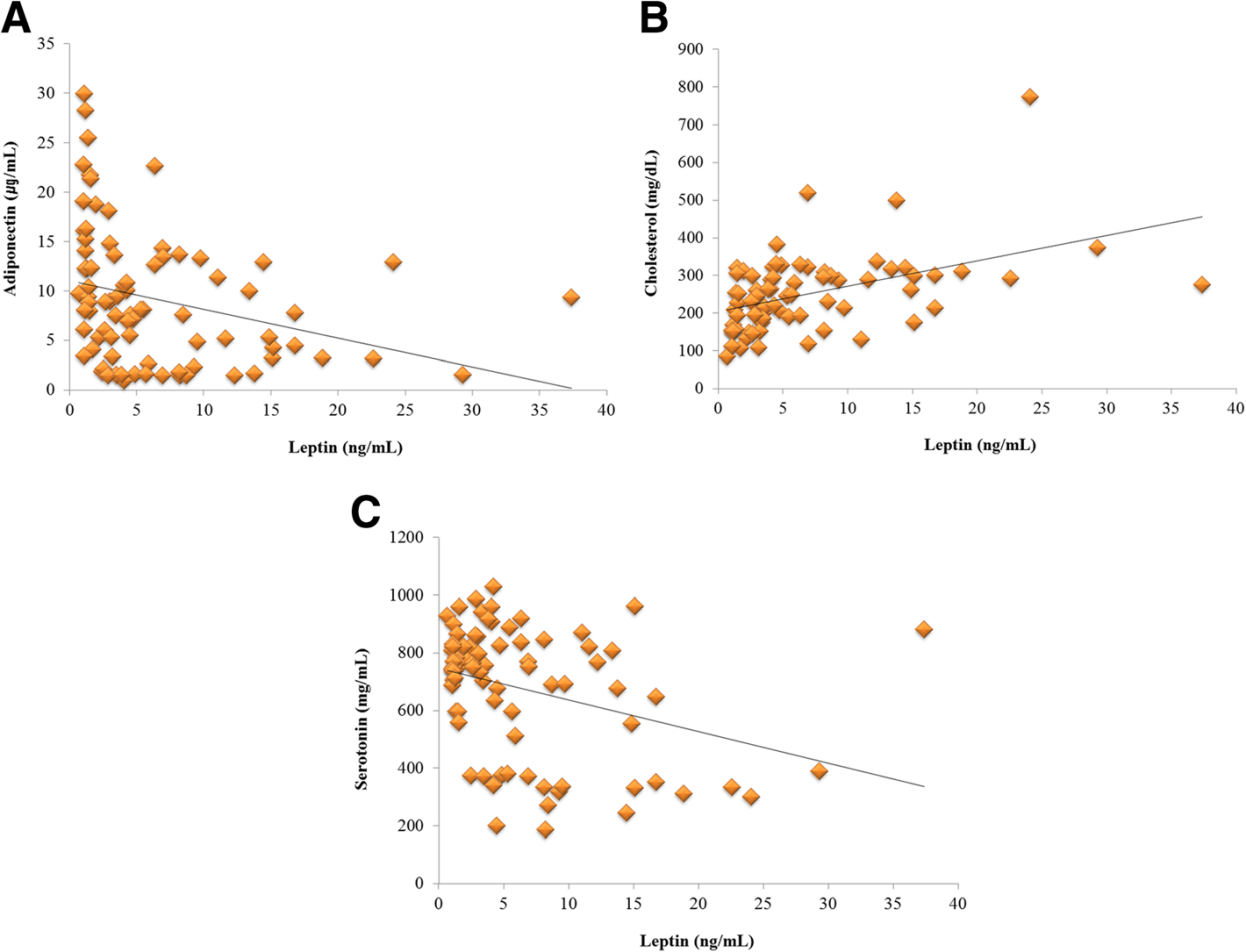
**Correlation among leptin, adiponectin, cholesterol and serotonin. (A)** Leptin and adiponectin showed negative correlation (*r* = -0.294, p < 0.01). **(B)** Leptin and Cholesterol showed positive correlation (*r* = 0.516, p < 0.01). **(C)** Leptin and serotonin showed negative correlation (*r* = -0.343, p < 0.01).

Leptin (*r* = 0.628, p < 0.01), TG (*r* = 0.491, p < 0.01) and cholesterol (*r* = 0.419, p < 0.01) levels were positively correlated with BCS and adiponectin (*r* = -0.446, p < 0.01) and 5HT (*r* = -0.490; p < 0.01) levels were negatively correlated with BCS (Figure 2). Leptin was negatively correlated with adiponectin (*r* = -0.294; p < 0.01) and 5HT (*r* = -0.343; p < 0.01), but leptin was positively correlated with cholesterol (*r* = 0.516; p < 0.01) (Figure 3).

## Discussion

In the current research, we found that obese dogs had lower 5HT and adiponectin levels and higher leptin levels than lean dogs. TG and cholesterol levels were high in the obese group compared with the lean group. Body fat is no longer considered simply as energy storage. Adipocytes produce many cytokines and hormones referred to as fat-derived peptides [12]. Adipokines include adiponectin, leptin, resistin, amyloid A, transforming growth factor β, tumor necrosis factor and interleukin-6 [7,12,19]. Currently, there is much interest in the role of adipokines in canine obesity [19]. Circulating leptin levels are positively correlated with adipose tissue mass, and exogenous leptin replacement decreases fast-induced hyperphagia [11]. However, the majority of obese animals and humans have high serum leptin levels, which suggests leptin resistance [11]. In this study, the serum leptin levels of dogs in the obese group were higher than those in the lean group, and adiponectin levels in the obese group were lower than those in the lean group (Table 2). Obesity can be a cause of leptin resistance, but a lack of sensitivity to circulating leptin may also induce obesity [11,20].

Middle-aged neutered male cats and middle-aged spayed female dogs have a high risk of being overweight or obese [8,21]. With aging, lean body mass gradually declines, resulting in a reduced basal metabolic rate (BMR) and reduced total daily energy needs. If accompanied by reduced exercise, the loss of lean body mass will be exacerbated. This study revealed no significant differences in obesity-related parameters, with the exception of cholesterol, between young and old dogs. However, spayed female dogs had a significantly higher BCS than intact females and this is the same result as a previous study [8]. Neutering decreases the BMR by 25% to 33% [22]. Most dog owners are unaware of the altered metabolic activity after ovariohysterectomy and do not decrease the food supply, which leads to increased food intake in dogs. This feeding behavior is often accompanied by decreased physical activity and, consequently, spayed female dogs gain weight easily.

In our study, the obese group had higher TG and cholesterol levels than the lean group. However, in the obese group, the average TG level (138.64) did not exceed 1000 mg/dL and the average cholesterol level (289.97) was less than 750 mg/dL. The goal of hyperlipidemia treatment is to maintain plasma lipid concentrations under a level at which health problems are likely to occur. Dietary intervention is recommended in dogs that have fasting TG levels greater than 500 mg/dL or cholesterol levels over 750 mg/dL [23]. In the present study, the obese group had high TG and cholesterol levels, but the likelihood of health problems induced by hyperlipidemia in these animals is marginal. In this study, the peripheral 5HT levels in the obese group were significantly lower than in the lean group, which is similar to human study results [13]. Furthermore, a negative correlation between leptin and 5HT was observed in this study. In the obese group, spayed female showed lower 5HT levels compared with intact female. In human studies, natural postmenopausal and ovariectomised women had lower 5HT levels than regularly menstruating women [24]. Estrogen withdrawal alters serotonergic functioning and exogenous estrogen supplementation could increase 5HT levels in postmenopausal women [15,24]. As with human studies, we found a similar phenomenon in spayed obese dogs. 5HT throughout the body influences food consumption by controlling satiety [25]. Therefore, low levels of 5HT could be a risk factor for obesity due to increased appetite. 5HT has a hypophagic effect in the CNS, and 5HT concentrations in the peripheral nervous system may not necessarily equate to 5HT levels or availability in the brain, since 5HT cannot cross the blood–brain barrier [14]. L-tryptophan, an amino acid and precursor to serotonin is converted to 5-hydroxy-L-tryptophan (5-HTP) and then to serotonin in both the CNS and PNS [26]. Brain tryptophan and serotonin levels are determined by the ratio of plasma tryptophan to other large neutral amino acids (LNAAs), which compete with tryptophan for uptake into the brain [14]. After eating carbohydrates, insulin is released, which promotes uptake of the LNAAs, but not tryptophan, into skeletal muscles. This helps tryptophan pass more easily into the brain, which increases serotonin production in the brain [14,25]. Increased 5HT production in the brain from carbohydrate-rich diets can induce mood-enhancing post-ingestion effects that motivate intake of such foods and, consequently, promote weight gain [14]. Circulating leptin interacts with peripheral 5HT and decreases appetite [3]. One mouse model study reported that plasma leptin was reduced by 5HT, and 5HT exerted a direct effect on adipocytes and regulated leptin release from adipocytes [15]. Moreover, 5HT is able to down-regulate adiponectin in the mouse adipocyte cell line [27]. Peripheral 5HT concentration of the obese group is significantly lower than the lean group in this study, and this is similar with human study results. Enterochromaffin (EC) cells in the intestinal epithelium release 5HT according to mechanical stimulation, to promote transit [28]. Experimentally a diet-induced obesity model showed decreased 5HT levels with a decreased number of EC cells [11]. The inflammation associated with changes in the GI microbiota is considered as the reason for decreased 5HT availability in obese status [28,29]. 5HT, as a neurotransmitter, controls food satiety, and, therefore, high 5HT concentrations decrease leptin and adiponectin concentrations. We found that 5HT is negatively correlated to leptin. Based on the previous result of a mouse model that 5HT could reduce the secretion of leptin, we can consider the possibility that lowered 5HT failed to properly suppress increasing secretion of leptin [15]. As seen in Figure 3C, however, there are more dogs presenting a high level of 5HT at a low level of leptin than ones presenting a low level of 5HT at a high level of leptin. Therefore, further studies would be needed with a larger number of dogs to evaluate the interaction between 5HT and leptin. The low level of adiponectin in the obese group was similar to the results of previous studies [30,31]. Adiponectin was negatively correlated with obesity [32]. The decreased adiponectin level in obesity is more significant in visceral than subcutaneous adiposity in humans, and the composition of adiponectin also changes with location in the body. 5HT suppresses adiponectin, and, therefore, a high peripheral adiponectin level can induce a low 5HT level. However, we observed low levels of both 5HT and adiponectin in the obese group in our study.

Through this research, we found that the level of peripheral 5HT is low in the obese group. Since 5HT is related to intestinal mobility, we can expect that a lower level of 5HT could reduce intestinal mobility. The reduced mobility, in turn, could allow gut microorganism to undergo energy harvesting for a longer period, which consequentially could aggravate obesity. Based on this hypothesis, further research is needed to identify that 5HT treatment could actually activate intestinal mobility and how it might work to reduce obesity. It is possible that humans are becoming obese as a result of overeating in order to maintain serotonin levels and the resulting positive mood. Therefore, serotonin agonists may be a treatment option for obesity in humans [33]. From this research, we found that the 5HT level of obese dogs was lower than that of lean dogs, therefore, we could assume that a serotonin agonist might be helpful in increasing the 5HT level in obese dogs. Increased 5HT levels via a serotonin agonist may control appetite and prompt intestinal motility. In human medicine, high levels of 5HT may be dangerous, and is known as serotonin syndrome [34]. Unfortunately, there have been few studies on the relationship between obesity and 5HT in the veterinary field so far. As obesity is considered to cause several diseases, based on the results of our research, more studies are needed to determine the impact and the mechanism of 5HT on leptin and adiponectin. Furthermore, as a treatment option for obesity in veterinary medicine, clinical trials of a 5HT agonist and considerations of adverse effects should be pursued.

There are a few limitations to this study. First of all, concerning the selection of the dogs in this study, the distribution of dogs does not balance between intact and castrated males. For example, there was a relatively fewer number of intact males than castrated males in the obese group. In addition, even though we identified several corrections among adipokines, 5HT, and other obesity-related parameters, we did not go further to formulate the mechanism of interactions among them. Although we found a significant difference in 5HT levels between the lean and obese groups, there was also variation within the groups. We tried to control for factors that influence 5HT levels such as platelet, diarrhea and food. However, we could not control emotion, social situation or the rhythm activity of an enrolled subject.

## Conclusions

5HT is an important appetite control neurotransmitter, but there are limited studies regarding 5HT levels related to obesity in veterinary medicine. Overall, we found that peripheral 5HT levels were lower in obese dogs than lean dogs, and 5HT was negatively correlated with BCS and leptin levels. To the best of our knowledge, this is the first study to evaluate peripheral 5HT levels in obese dogs.

## Competing interests

The authors declare that they have no competing interests.

## Authors’ contributions

HJP, KWS and KHS participated in the conception and design of this study. HJP and SEL participated in sample collection, laboratory testing and drafting of article. JHO, KWS and HJP participated in statistical analysis. KWS, KHS, and SEL participated in critical revisions. All authors read and approved the final manuscript.
